# Supplementary material for: Hot spot prediction in protein-protein interactions by an ensemble system
Source: BMC Syst Biol. 2018 Dec 31;12(Suppl 9):132. doi: 10.1186/s12918-018-0665-8 (PMC6311905; doi:10.1186/s12918-018-0665-8)
Supplement: Supplementary file 2 — All relevant MATLAB code. A simple Matlab implement of our predictor. (ZIP 19,742 kb) [file 12918_2018_665_MOESM2_ESM.zip › Additional file 2/Read ASA/ASA website/dbMPIKT/1YY9C.html]

 NetSurfP - Protein Surface Accessibility and Secondary Structure Predictions

  

|  |  |  |
| --- | --- | --- |
|  |  | NetSurfP - Protein Surface Accessibility and Secondary Structure Predictions  Technical University of Denmark |

  


---

```
# For publication of results, please cite:
# A generic method for assignment of reliability scores applied to solvent accessibility predictions.
# Bent Petersen, Thomas Nordahl Petersen, Pernille Andersen, Morten Nielsen and Claus Lundegaard
# BMC Structural Biology 2009, 9:51 doi:10.1186/1472-6807-9-51
#
# Column 1: Class assignment - B for buried or E for Exposed - Threshold: 25% exposure, but not based on RSA
# Column 2: Amino acid
# Column 3: Sequence name
# Column 4: Amino acid number
# Column 5: Relative Surface Accessibility - RSA
# Column 6: Absolute Surface Accessibility
# Column 7: Z-fit score for RSA prediction
# Column 8: Probability for Alpha-Helix
# Column 9: Probability for Beta-strand
# Column 10: Probability for Coil
E D  1YY9C                  1    0.794 114.401   0.643   0.003   0.003   0.994
B I  1YY9C                  2    0.214  39.627  -0.081   0.003   0.718   0.279
E L  1YY9C                  3    0.428  78.422   1.427   0.001   0.959   0.040
B L  1YY9C                  4    0.126  23.089   0.858   0.001   0.900   0.099
E T  1YY9C                  5    0.442  61.375   0.542   0.002   0.816   0.182
B Q  1YY9C                  6    0.177  31.576   0.553   0.004   0.514   0.481
E S  1YY9C                  7    0.349  40.891   0.118   0.004   0.138   0.858
E P  1YY9C                  8    0.458  64.948  -0.915   0.018   0.019   0.964
E V  1YY9C                  9    0.528  81.154  -1.021   0.018   0.088   0.893
E I  1YY9C                 10    0.338  62.438  -1.293   0.021   0.451   0.528
B L  1YY9C                 11    0.247  45.152  -0.166   0.021   0.756   0.223
E S  1YY9C                 12    0.478  56.057  -0.677   0.018   0.846   0.136
B V  1YY9C                 13    0.200  30.771   0.191   0.021   0.756   0.223
E S  1YY9C                 14    0.412  48.286   0.282   0.004   0.420   0.576
E P  1YY9C                 15    0.508  72.128  -0.029   0.004   0.085   0.910
E G  1YY9C                 16    0.671  52.784  -1.094   0.005   0.015   0.979
E E  1YY9C                 17    0.497  86.756   0.355   0.004   0.138   0.858
E R  1YY9C                 18    0.539 123.523   0.957   0.004   0.616   0.381
B V  1YY9C                 19    0.065   9.929  -0.017   0.001   0.900   0.099
E S  1YY9C                 20    0.437  51.181   1.469   0.001   0.959   0.040
B F  1YY9C                 21    0.052  10.336   0.275   0.001   0.959   0.040
E S  1YY9C                 22    0.365  42.743   0.992   0.001   0.900   0.099
B C  1YY9C                 23    0.028   3.987   0.169   0.001   0.900   0.099
E R  1YY9C                 24    0.411  94.211   0.900   0.001   0.900   0.099
B A  1YY9C                 25    0.175  19.296  -0.964   0.002   0.816   0.182
E S  1YY9C                 26    0.356  41.711  -0.846   0.004   0.616   0.381
E Q  1YY9C                 27    0.590 105.392  -2.601   0.004   0.085   0.910
E S  1YY9C                 28    0.445  52.189  -2.338   0.005   0.045   0.951
E I  1YY9C                 29    0.492  91.057  -1.432   0.004   0.085   0.910
E G  1YY9C                 30    0.426  33.518  -1.791   0.004   0.138   0.858
B T  1YY9C                 31    0.239  33.191  -0.740   0.005   0.262   0.733
B N  1YY9C                 32    0.141  20.672   0.253   0.003   0.718   0.279
B I  1YY9C                 33    0.027   5.013   0.444   0.001   0.900   0.099
B H  1YY9C                 34    0.061  11.096   0.224   0.001   0.900   0.099
B W  1YY9C                 35    0.027   6.566   0.590   0.011   0.918   0.071
B Y  1YY9C                 36    0.054  11.540   0.686   0.001   0.959   0.040
B Q  1YY9C                 37    0.169  30.201   0.243   0.001   0.900   0.099
B Q  1YY9C                 38    0.151  27.004   0.294   0.004   0.616   0.381
E R  1YY9C                 39    0.423  96.913   1.375   0.004   0.138   0.858
E T  1YY9C                 40    0.569  78.865  -0.584   0.005   0.015   0.979
E N  1YY9C                 41    0.772 112.948  -2.161   0.003   0.003   0.994
E G  1YY9C                 42    0.549  43.238   0.083   0.018   0.047   0.935
E S  1YY9C                 43    0.349  40.879  -0.380   0.018   0.088   0.893
B P  1YY9C                 44    0.184  26.095  -1.096   0.021   0.279   0.699
E R  1YY9C                 45    0.378  86.470  -0.175   0.004   0.616   0.381
B L  1YY9C                 46    0.201  36.785   0.324   0.003   0.718   0.279
B L  1YY9C                 47    0.089  16.259  -0.751   0.001   0.900   0.099
B I  1YY9C                 48    0.072  13.412  -0.789   0.001   0.900   0.099
B K  1YY9C                 49    0.140  28.839  -0.568   0.001   0.900   0.099
B Y  1YY9C                 50    0.180  38.381  -1.622   0.002   0.816   0.182
B A  1YY9C                 51    0.158  17.390  -1.712   0.004   0.420   0.576
B S  1YY9C                 52    0.270  31.621  -1.446   0.020   0.205   0.775
E E  1YY9C                 53    0.588 102.724  -1.722   0.018   0.088   0.893
E S  1YY9C                 54    0.457  53.560  -0.805   0.018   0.047   0.935
E I  1YY9C                 55    0.581 107.559  -1.907   0.018   0.019   0.964
E S  1YY9C                 56    0.686  80.364  -1.292   0.018   0.047   0.935
E G  1YY9C                 57    0.608  47.889  -1.232   0.018   0.088   0.893
B I  1YY9C                 58    0.239  44.178  -0.212   0.018   0.088   0.893
E P  1YY9C                 59    0.563  79.890  -1.119   0.018   0.088   0.893
E S  1YY9C                 60    0.660  77.375  -1.746   0.004   0.085   0.910
E R  1YY9C                 61    0.397  90.959  -0.933   0.005   0.262   0.733
E F  1YY9C                 62    0.373  74.921  -1.398   0.004   0.514   0.481
E S  1YY9C                 63    0.304  35.594  -2.040   0.004   0.514   0.481
B G  1YY9C                 64    0.296  23.264  -2.804   0.004   0.616   0.381
E S  1YY9C                 65    0.444  52.072  -1.273   0.004   0.616   0.381
E G  1YY9C                 66    0.481  37.894  -1.172   0.004   0.514   0.481
E S  1YY9C                 67    0.514  60.299  -2.093   0.005   0.262   0.733
E G  1YY9C                 68    0.575  45.252  -1.724   0.004   0.138   0.858
E T  1YY9C                 69    0.425  58.934  -0.968   0.005   0.336   0.660
E D  1YY9C                 70    0.346  49.887  -0.066   0.002   0.816   0.182
B F  1YY9C                 71    0.083  16.618  -0.025   0.001   0.900   0.099
B T  1YY9C                 72    0.205  28.420   0.235   0.001   0.900   0.099
B L  1YY9C                 73    0.038   6.939  -0.154   0.001   0.900   0.099
B S  1YY9C                 74    0.142  16.689  -0.455   0.001   0.900   0.099
B I  1YY9C                 75    0.042   7.751  -0.027   0.001   0.900   0.099
E N  1YY9C                 76    0.271  39.674   0.335   0.002   0.816   0.182
B S  1YY9C                 77    0.269  31.515  -1.482   0.022   0.552   0.426
B V  1YY9C                 78    0.248  38.071  -0.479   0.020   0.205   0.775
E E  1YY9C                 79    0.441  77.113   0.413   0.019   0.141   0.840
E S  1YY9C                 80    0.391  45.802  -0.873   0.020   0.205   0.775
E E  1YY9C                 81    0.573 100.173  -1.926   0.019   0.141   0.840
E D  1YY9C                 82    0.558  80.379  -0.713   0.018   0.088   0.893
E I  1YY9C                 83    0.419  77.441  -1.309   0.019   0.141   0.840
E A  1YY9C                 84    0.394  43.397  -0.610   0.005   0.262   0.733
E D  1YY9C                 85    0.470  67.741   0.600   0.003   0.718   0.279
B Y  1YY9C                 86    0.096  20.537   0.316   0.002   0.816   0.182
B Y  1YY9C                 87    0.188  40.218   0.513   0.002   0.816   0.182
B C  1YY9C                 88    0.116  16.329  -1.106   0.003   0.718   0.279
B Q  1YY9C                 89    0.198  35.416   0.024   0.022   0.552   0.426
B Q  1YY9C                 90    0.159  28.433  -1.268   0.022   0.359   0.619
B N  1YY9C                 91    0.304  44.535  -1.550   0.022   0.359   0.619
E N  1YY9C                 92    0.338  49.556  -1.686   0.022   0.359   0.619
E N  1YY9C                 93    0.350  51.196  -1.480   0.064   0.216   0.721
E W  1YY9C                 94    0.395  94.998  -1.404   0.056   0.142   0.802
E P  1YY9C                 95    0.321  45.493  -1.562   0.052   0.084   0.864
E T  1YY9C                 96    0.371  51.472  -1.366   0.052   0.084   0.864
E T  1YY9C                 97    0.384  53.261  -1.011   0.056   0.142   0.802
B F  1YY9C                 98    0.242  48.489  -1.576   0.056   0.142   0.802
E G  1YY9C                 99    0.365  28.702  -0.929   0.056   0.142   0.802
E A  1YY9C                100    0.366  40.366  -0.900   0.056   0.142   0.802
E G  1YY9C                101    0.358  28.175  -1.210   0.064   0.216   0.721
E T  1YY9C                102    0.395  54.745  -1.633   0.064   0.216   0.721
E K  1YY9C                103    0.339  69.691  -1.270   0.022   0.359   0.619
E L  1YY9C                104    0.375  68.608  -1.168   0.022   0.359   0.619
E E  1YY9C                105    0.324  56.673  -0.916   0.022   0.359   0.619
E L  1YY9C                106    0.367  67.143  -1.636   0.022   0.359   0.619
E K  1YY9C                107    0.356  73.229  -1.279   0.022   0.359   0.619
E R  1YY9C                108    0.352  80.516  -1.438   0.022   0.359   0.619
E T  1YY9C                109    0.456  63.317  -1.598   0.022   0.359   0.619
E V  1YY9C                110    0.447  68.781  -1.275   0.021   0.279   0.699
E A  1YY9C                111    0.418  46.097  -1.235   0.020   0.205   0.775
E A  1YY9C                112    0.373  41.149  -1.196   0.018   0.088   0.893
E P  1YY9C                113    0.347  49.168  -1.796   0.052   0.084   0.864
E S  1YY9C                114    0.446  52.224  -1.098   0.056   0.142   0.802
B V  1YY9C                115    0.304  46.802  -1.360   0.064   0.216   0.721
E F  1YY9C                116    0.427  85.679  -1.344   0.135   0.317   0.548
E I  1YY9C                117    0.372  68.894  -1.689   0.135   0.317   0.548
E F  1YY9C                118    0.395  79.357  -1.583   0.135   0.317   0.548
E P  1YY9C                119    0.394  55.980  -1.034   0.135   0.317   0.548
E P  1YY9C                120    0.430  60.989  -1.345   0.125   0.227   0.648
E S  1YY9C                121    0.399  46.810  -1.127   0.125   0.227   0.648
E D  1YY9C                122    0.440  63.418  -1.507   0.118   0.150   0.732
E E  1YY9C                123    0.461  80.519  -1.597   0.118   0.150   0.732
E Q  1YY9C                124    0.400  71.440  -1.951   0.113   0.087   0.800
E L  1YY9C                125    0.459  84.025  -1.351   0.113   0.087   0.800
E K  1YY9C                126    0.417  85.818  -1.082   0.053   0.043   0.903
E S  1YY9C                127    0.533  62.526  -1.596   0.018   0.088   0.893
E G  1YY9C                128    0.576  45.363  -1.590   0.018   0.088   0.893
E T  1YY9C                129    0.385  53.399  -0.373   0.004   0.197   0.799
B A  1YY9C                130    0.077   8.441  -1.104   0.004   0.514   0.481
B S  1YY9C                131    0.159  18.576   0.266   0.002   0.816   0.182
B V  1YY9C                132    0.030   4.657   0.366   0.001   0.900   0.099
B V  1YY9C                133    0.046   7.086   0.158   0.001   0.959   0.040
B C  1YY9C                134    0.016   2.204   1.163   0.001   0.900   0.099
B L  1YY9C                135    0.130  23.785   0.279   0.001   0.900   0.099
B L  1YY9C                136    0.035   6.445  -1.407   0.002   0.816   0.182
E N  1YY9C                137    0.336  49.146   0.150   0.004   0.514   0.481
E N  1YY9C                138    0.500  73.215  -1.414   0.005   0.262   0.733
E F  1YY9C                139    0.522 104.685  -1.404   0.005   0.262   0.733
E Y  1YY9C                140    0.302  64.580  -0.973   0.005   0.262   0.733
E P  1YY9C                141    0.415  58.818  -1.881   0.004   0.197   0.799
E R  1YY9C                142    0.590 135.202  -2.326   0.004   0.138   0.858
E E  1YY9C                143    0.463  80.851  -0.989   0.004   0.420   0.576
B A  1YY9C                144    0.148  16.310   0.193   0.003   0.718   0.279
B K  1YY9C                145    0.273  56.218   0.267   0.001   0.900   0.099
B V  1YY9C                146    0.042   6.394  -0.072   0.001   0.900   0.099
B Q  1YY9C                147    0.189  33.827   0.235   0.011   0.918   0.071
B W  1YY9C                148    0.052  12.458   0.422   0.011   0.918   0.071
B K  1YY9C                149    0.202  41.572   0.907   0.001   0.900   0.099
E V  1YY9C                150    0.373  57.361   0.237   0.002   0.816   0.182
E D  1YY9C                151    0.543  78.217   0.121   0.021   0.279   0.699
E N  1YY9C                152    0.629  92.130  -0.704   0.018   0.047   0.935
E A  1YY9C                153    0.653  71.928  -0.638   0.004   0.085   0.910
E L  1YY9C                154    0.685 125.387  -1.889   0.004   0.197   0.799
E Q  1YY9C                155    0.436  77.834  -0.125   0.004   0.197   0.799
E S  1YY9C                156    0.453  53.045  -1.404   0.004   0.138   0.858
E G  1YY9C                157    0.604  47.535  -1.875   0.019   0.141   0.840
E N  1YY9C                158    0.529  77.475  -1.795   0.020   0.205   0.775
E S  1YY9C                159    0.446  52.259  -1.168   0.022   0.552   0.426
E Q  1YY9C                160    0.373  66.636  -0.071   0.023   0.655   0.322
E E  1YY9C                161    0.527  92.137  -1.493   0.021   0.451   0.528
E S  1YY9C                162    0.457  53.549  -2.101   0.021   0.279   0.699
E V  1YY9C                163    0.444  68.274  -1.472   0.021   0.279   0.699
E T  1YY9C                164    0.325  45.119  -0.594   0.021   0.279   0.699
E E  1YY9C                165    0.317  55.397  -1.701   0.021   0.279   0.699
E Q  1YY9C                166    0.576 102.838  -1.726   0.022   0.359   0.619
E D  1YY9C                167    0.471  67.900  -1.499   0.005   0.262   0.733
E S  1YY9C                168    0.295  34.574  -1.903   0.004   0.197   0.799
E K  1YY9C                169    0.655 134.631  -1.433   0.005   0.045   0.951
E D  1YY9C                170    0.760 109.502  -2.574   0.003   0.003   0.994
E S  1YY9C                171    0.488  57.240  -2.049   0.005   0.045   0.951
E T  1YY9C                172    0.364  50.528  -0.418   0.004   0.420   0.576
B Y  1YY9C                173    0.162  34.577  -1.519   0.004   0.616   0.381
B S  1YY9C                174    0.112  13.150  -1.979   0.002   0.816   0.182
B L  1YY9C                175    0.106  19.390  -1.226   0.001   0.900   0.099
B S  1YY9C                176    0.176  20.627  -0.822   0.001   0.959   0.040
B S  1YY9C                177    0.039   4.629  -1.182   0.001   0.959   0.040
B T  1YY9C                178    0.171  23.718   0.025   0.001   0.959   0.040
B L  1YY9C                179    0.040   7.287  -0.342   0.001   0.959   0.040
E T  1YY9C                180    0.314  43.552   0.770   0.001   0.900   0.099
B L  1YY9C                181    0.066  12.121   0.425   0.002   0.816   0.182
E S  1YY9C                182    0.376  44.091   1.195   0.022   0.552   0.426
E K  1YY9C                183    0.397  81.704  -0.738   0.022   0.359   0.619
E A  1YY9C                184    0.549  60.500  -0.735   0.021   0.279   0.699
E D  1YY9C                185    0.524  75.465   0.843   0.021   0.279   0.699
B Y  1YY9C                186    0.209  44.770  -0.524   0.019   0.141   0.840
E E  1YY9C                187    0.652 113.922   0.585   0.004   0.085   0.910
E K  1YY9C                188    0.667 137.161   0.582   0.005   0.045   0.951
E H  1YY9C                189    0.629 114.470  -0.085   0.004   0.085   0.910
E K  1YY9C                190    0.439  90.241   0.212   0.004   0.420   0.576
E V  1YY9C                191    0.455  69.903   1.579   0.002   0.816   0.182
B Y  1YY9C                192    0.064  13.655   0.938   0.001   0.959   0.040
E A  1YY9C                193    0.317  34.900   1.692   0.001   0.959   0.040
B C  1YY9C                194    0.026   3.721   0.349   0.001   0.959   0.040
B E  1YY9C                195    0.266  46.488   1.182   0.001   0.959   0.040
B V  1YY9C                196    0.102  15.631   0.343   0.001   0.900   0.099
E T  1YY9C                197    0.435  60.334   0.522   0.001   0.900   0.099
B H  1YY9C                198    0.167  30.414   0.032   0.002   0.816   0.182
E Q  1YY9C                199    0.630 112.500   0.146   0.005   0.336   0.660
E G  1YY9C                200    0.550  43.254  -1.671   0.004   0.085   0.910
E L  1YY9C                201    0.359  65.660  -0.499   0.005   0.045   0.951
E S  1YY9C                202    0.642  75.219  -0.299   0.005   0.045   0.951
E S  1YY9C                203    0.539  63.171   0.160   0.018   0.047   0.935
E P  1YY9C                204    0.455  64.536   0.072   0.019   0.141   0.840
B V  1YY9C                205    0.194  29.741   0.195   0.022   0.552   0.426
E T  1YY9C                206    0.553  76.687   0.462   0.002   0.816   0.182
E K  1YY9C                207    0.271  55.786   1.030   0.001   0.900   0.099
E S  1YY9C                208    0.474  55.529   0.157   0.001   0.900   0.099
B F  1YY9C                209    0.173  34.781   0.044   0.011   0.918   0.071
E N  1YY9C                210    0.472  69.101   0.692   0.003   0.718   0.279
E R  1YY9C                211    0.473 108.386   0.154   0.020   0.205   0.775
E G  1YY9C                212    0.616  48.487  -1.841   0.018   0.047   0.935
E A  1YY9C                213    0.792  87.278  -1.085   0.003   0.003   0.994

Explain the output.  Go back.
```

---
